# Enzyme Responsive Vaginal Microbicide Gels Containing Maraviroc and Tenofovir Microspheres Designed for Acid Phosphatase-Triggered Release for Pre-Exposure Prophylaxis of HIV-1: A Comparative Analysis of a Bigel and Thermosensitive Gel

**DOI:** 10.3390/gels8010015

**Published:** 2021-12-24

**Authors:** Sabdat Ozichu Ekama, Margaret O. Ilomuanya, Chukwuemeka Paul Azubuike, James Babatunde Ayorinde, Oliver Chukwujekwu Ezechi, Cecilia Ihuoma Igwilo, Babatunde Lawal Salako

**Affiliations:** 1 Department of Pharmaceutics and Pharmaceutical Technology, Faculty of Pharmacy, University of Lagos, Surulere, Lagos P.M.B 12003, Nigeria; milomuanya@unilag.edu.ng (M.O.I.); cazubuike@unilag.edu.ng (C.P.A.); cigwilo@unilag.edu.ng (C.I.I.); 2 Nigerian Institute of Medical Research, 6 Edmund Crescent, Yaba, Lagos P.M.B 12003, Nigeria; ayorindejames@gmail.com (J.B.A.); oezechi@nimr.gov.ng (O.C.E.); tundesalako@nimr.gov.ng (B.L.S.)

**Keywords:** HIV prevention, pre-exposure prophylaxis, HIV/AIDS, vaginal gels microbicides, acid phosphatase, microparticles

## Abstract

The challenges encountered with conventional microbicide gels has necessitated the quest for alternative options. This study aimed to formulate and evaluate a bigel and thermosensitive gel, designed to combat the challenges of leakage and short-residence time in the vagina. Ionic-gelation technique was used to formulate maraviroc and tenofovir microspheres. The microspheres were incorporated into a thermosensitive gel and bigel, then evaluated. Enzyme degradation assay was used to assess the effect of the acid phosphatase enzyme on the release profile of maraviroc and tenofovir microspheres. HIV efficacy and cytotoxicity of the microspheres were assessed using HIV-1-BaL virus strain and HeLa cell lines, respectively. Maraviroc and tenofovir release kinetics followed zero-order and Higuchi model kinetics. However, under the influence of the enzyme, maraviroc release was governed by first-order model, while tenofovir followed a super case II transport-mechanism. The altered mode of release and drug transport mechanism suggests a triggered release. The assay of the microspheres suspension on the HeLa cells did not show signs of cytotoxicity. The thermosensitive gel and bigel elicited a progressive decline in HIV infectivity, until at concentrations of 1 μg/mL and 0.1 μg/mL, respectively. The candidate vaginal gels have the potential for a triggered release by the acid phosphatase enzyme present in the seminal fluid, thus, serving as a strategic point to prevent HIV transmission.

## 1. Introduction

Preventing new human immunodeficiency virus (HIV) infections using microbicides as a pre-exposure prophylactic measure has gained attention in recent times because of the disproportionate prevalence among women globally. HIV microbicides are medicaments, containing antiretroviral drugs that are administered via the vagina or rectum to prevent HIV infection.

Male to female heterosexual intercourse is a major mode of HIV transmission, and this has necessitated a focus on prevention strategies targeting viral infection at the point of sexual intercourse [1,2].

Pre-exposure prophylaxis is the use of antiretroviral drugs among HIV negative individuals to prevent HIV infection before possible contact with an infected individual. This is commonly offered to individuals at high risk of HIV infection, such as couples in a serodiscordant relationship [3].

Microbicides are dosage forms used to administer pre-exposure prophylactic treatment. They have been designed to target HIV at the point of sexual intercourse, using different mechanisms; however, the early first-generation vaginal microbicide gels (SAVVY, PRO 2000, SLS, N-9) were non-specific in their mode of action on the HIV life cycle and were all ineffective in clinical trials [4].

The CAPRISA trial employed an antiretroviral drug, which is specific in its mode of action, formulated as a 1% tenofovir conventional microbicide gel. The trial reported a reduction in the risk of HIV acquisition by 39% among the study participants.

However, challenges associated with the conventional microbicide gel tested in the CAPRISA clinical trial, which include leakage, messiness, short vaginal residence time, and coital dependent use, which affected adherence to its use, as well as its efficacy, have necessitated the exploration of novel targeted and smart drug delivery techniques [5].

These novel drug delivery techniques are designed to target the active ingredients to the point of drug action, and one of the several ways to achieve targeted drug delivery is the use of microspheres (microparticles). They are free-flowing particles made from polymer, which could be modified to have mucoadhesive properties, such that it becomes adhesive to the vagina mucosa upon administration, thus increasing its residence time [6].

Stimuli sensitive systems, designed to trigger drug release in the presence of a stimulus, such as a change in pH, temperature, or enzymes, are also novel approaches to achieve smart drug delivery [7].

The stimulus of interest in this study is the enzyme acid phosphatase, which is a component of the male seminal fluid. Acid phosphatase is a glycoprotein with a molecular weight of 100,000 to 120,000, found in high concentrations in the male seminal fluid.

Seminal fluid is a rich source of enzymes, such as prostatic acid phosphatase and hyaluronidase. Prostatic acid phosphatase is an enzyme constituent of the seminal fluid, usually released from the prostate gland; it is also used as a tool in forensic examination to determine the presence of semen in cases of rape or sexual abuse [8,9].

Acid phosphatases are known to hydrolyze phosphate groups, and they are synthesized by epithelial cells that line the ductal elements within the prostrate. Therefore, its presence in the semen has been utilized in this study as a stimulus for a drug polymer microparticulate system that the enzyme can hydrolyze to give rise to polymer breakdown and a resultant drug release.

The candidate antiretroviral drugs, maraviroc and tenofovir, employed in this study are entry inhibitors and nucleotide reverse transcriptase inhibitors, respectively [10], which are designed and formulated as microspheres to be incorporated into a thermosensitive gel and bigel, which will serve as carriers for the microspheres. The polymer chitosan is a natural mucoadhesive polymer [11], which has been selected for the formulation of microspheres via a crosslinking process utilizing tripolyphosphate as a crosslinking agent.

These gels will be administered via the vaginal route to offer controlled and sustained release, with the goal of serving as a prophylactic agent against HIV-1 infection in women. Gels are of different types, the composition of the external liquid component, which could be either water or organic solvents, determines its classification, either as a hydrogel or organogel, respectively.

Thermosensitive gels are hydrogels that are designed to be liquid at room temperature but becomes viscous upon administration at vaginal temperature to enhance its retention, prevent leakage, and increase residence time [12].

Bigels are a hybrid of an organogel, and a hydrogel designed to allow for simultaneous incorporation of drugs that differ in hydrophilicity, which allows for better spreadability and retention [13,14].

This study seeks to explore the use of the polymer chitosan, crosslinked with tripolyphosphate, as a carrier for the antiretroviral drugs maraviroc and tenofovir, in the form of mucoadhesive microspheres. Furthermore, a stimulus-triggered release of the active ingredients is anticipated. This could occur because of the hydrolyzing effect of the acid phosphatase enzyme on the phosphate group of tripolyphosphate, leading to hydrolysis and breakdown of the microsphere, thus triggering the release of the drugs in the presence of semen during heterosexual intercourse. The hypothesis that a triggered release of the active drugs from the microspheres will occur in the presence of acid phosphatase enzyme was tested in this study.

This study, therefore, aims to formulate and evaluate vaginal microbicide gels containing maraviroc and tenofovir microspheres, designed for acid phosphatase-triggered release for pre-exposure prophylaxis of HIV-1.

## 2. Results and Discussion

### 2.1. Enzyme Degradation Assay

The cumulative percentage of drug released from the microspheres, under the influence of acid phosphatase enzyme and without it, is portrayed in Table 1, with the coefficient of correlation (R^2^) and drug transport mechanism (*n*) of the kinetic models shown in Table 2. In the presence of the enzyme, the release of microspheres containing maraviroc started after 12 h, but there was an observed increase in the percentage of drug released which stopped at the end of 72 h. In contrast to the scenario without the enzymes, the percentage of drug released was less, and drug release did not end at 72 h (Table 1).

**Figure 1:**
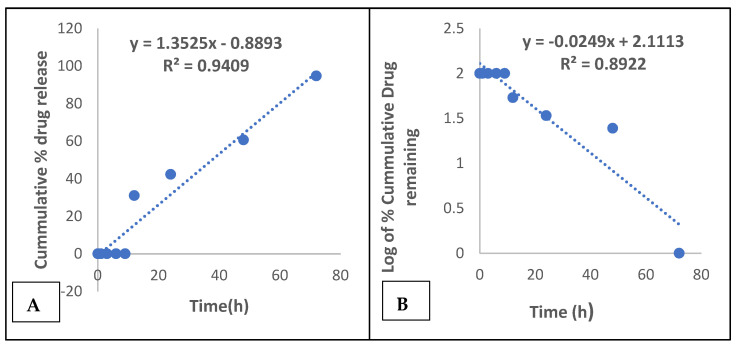
(**A**) Zero-order kinetic release of maraviroc from microspheres. (**B**) First-order kinetic release of maraviroc from microspheres facilitated with enzymes.

In the absence of the enzyme, maraviroc release from the microspheres followed a zero-order (R^2^ = 0.9409) kinetic release via a super case II transport mechanism (*n* = 1.1329), while under the influence of the enzyme it followed a first-order (R^2^ = 0.8922) kinetic release model via a super case II transport mechanism (*n* = 1.239), as shown in Figure 1A,B and Table 2. The amount of drug released for the microspheres containing tenofovir was higher, and its rate of release increased from 1 h till 24 h, under the influence of acid phosphatase enzyme, with a completion of drug release at 24 h.

**Figure 2:**
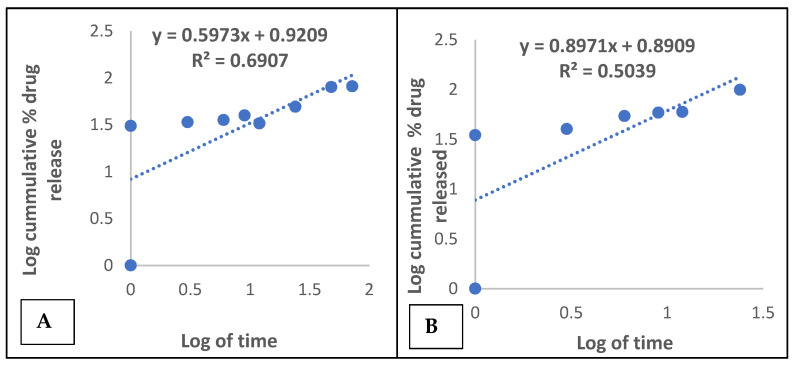
(**A**) Korsmeyer-peppas model kinetic release of tenofovir from microspheres. (**B**) Korsmeyer-peppas model kinetic release for tenofovir from microspheres facilitated with enzymes.

In comparison to the amount released without the enzymes, the percentage drugs released was less and drug release was not completed as at 72 h (Table 1). The release kinetic model for tenofovir followed a Higuchi model kinetics of release with and without the enzyme (Figure 2A,B); however, the release mechanism of transport was via a non-Fickian transport system without the enzyme (*n* = 0.5973) and a super case II transport mechanism (*n* = 0.8971) under the influence of the enzyme [15].

### 2.2. Characterization of Thermosensitive Gel Containing Maraviroc and Tenofovir

**Figure 3:**
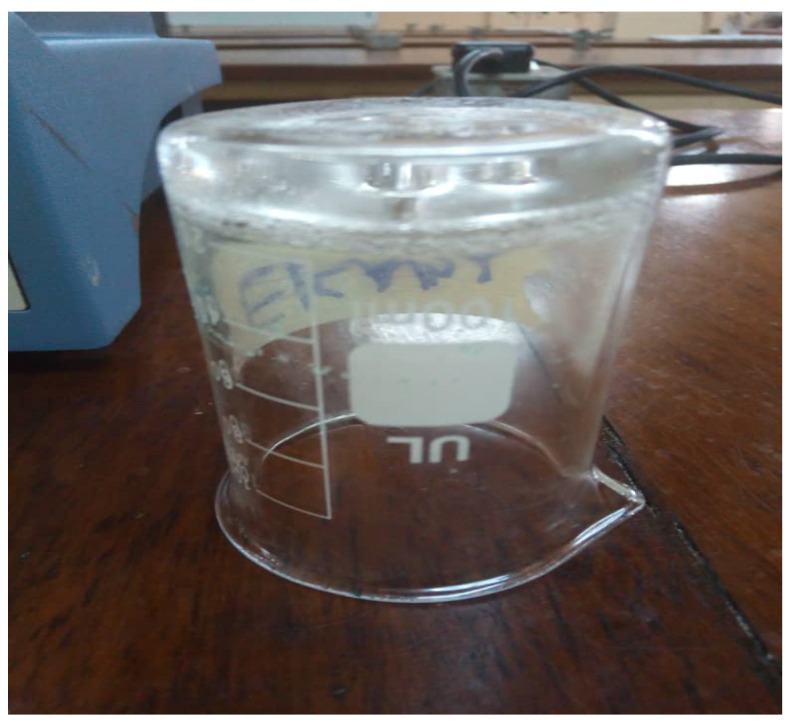
Thermosensitive gel, showing gelation at 36.4 °C.

The varying pluronic combinations had gelation temperatures varying from 33.4 °C to 42.6 °C, while combinations with pluronic F127 below 2g showed no gelation. The gelation time ranged from 37 to 120 s. The pH of the various gel combinations was acidic, ranging from 5.69 to 5.97 (Table 3). The optimal thermosensitive gel had a gelation temperature and time of 36.4 °C and 36 s, respectively, with a pH of 5.83 (Figure 3). The viscosity of the gel ranged from 24 cps and 88 cps to 62,887 cps at 4 °C, 25 °C, and 37 °C, respectively, with an osmolality value of 991 mOsm/kg (Table 4).

### 2.3. Characterization of the Organogel and Bigel

**Figure 4:**
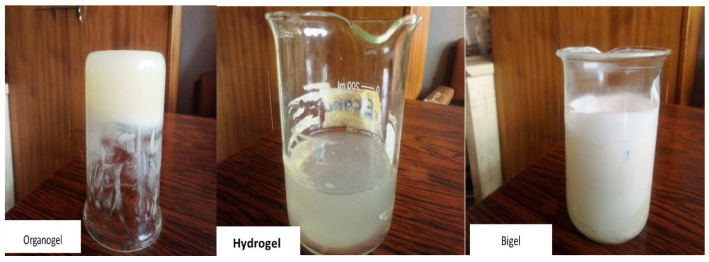
Organogel, hydrogel, and bigel mix.

The minimum gelation concentration of Span 60 in the organogel was 10%. Organogels were not formed at 2% or 5%. Furthermore, F3 was the optimal formulation selected for the preparation of the bigel (Table 5). The organogel and hydrogel formulations were combined at varying ratios to form a bigel mix, with a corresponding pH assessment (Table 6). The ratio 1:1 of organogel to hydrogel was used for the bigel formulation (Figure 4). The pH of the formed bigel was acidic, and its values ranged from 3.5–5.6. The optimal bigel had a pH of 3.65, with viscosity and osmolality of 8840 cps and 628 mOsm/kg, respectively (Table 7).

### 2.4. In-Vitro Cytotoxicity of Microspheres

Assuming a significance level (α) of 0.05 and 95% confidence interval, a two- sample *t*-test, comparing the means of absorbance between the test versus the controls, was conducted to test for significance (Table 8). The percentage viability of the cells exposed to the varying maraviroc/tenofovir concentrations ranged from 71.2% to 98.4%, which is a measure of the live HeLa cells. The HeLa cell exposed to the positive control had a percentage viability of 13.5%.

There was no statistically significant difference between the absorbance of the negative control and the maraviroc/tenofovir concentrations of 10 μg/mL, 1 μg/mL, and 0.1 μg/mL (*p* = 0.054, 0.069, and 0.098, respectively).

There was a statistically significant difference between the absorbance of the positive control and all varying concentrations of the maraviroc/tenofovir concentrations (*p* < 0.001).

**Figure 5:**
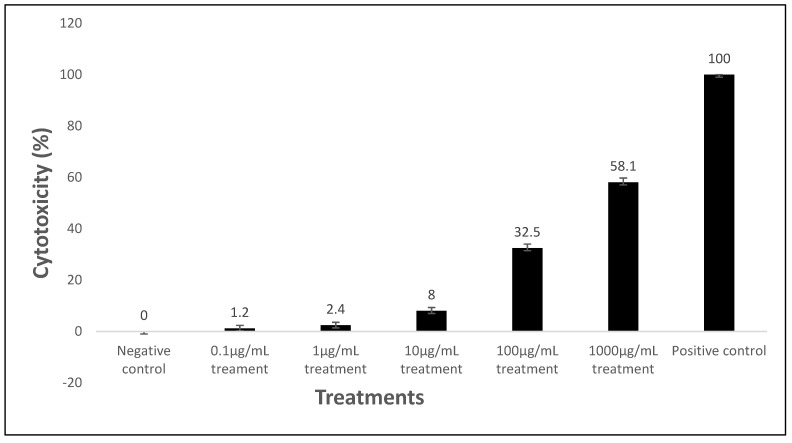
Cytotoxic effect of the varying concentrations of maraviroc and tenofovir microspheres, negative and positive control on the HeLa cell lines. Results are given as mean ± SD, *n* = 3.

Furthermore, as shown in Figure 5, the percentage cytotoxicity of the various maraviroc and tenofovir concentrations were minimal (<8%) from concentrations of 10 µg/mL and below, compared to higher concentrations and the positive control.

### 2.5. HIV Efficacy and TZM-bl Assay

Results from the in-vitro concentration response curve of the thermosensitive gel and bigel showed an initial threshold dose at a concentration of 0.0001g/mL. This indicates the onset of action of a decline in HIV infectivity, after which there was a progressive decline until at maximal concentration of 0.1 and 1.0 μg/mL, respectively.

There was a 10-fold difference between the maximal effective dose of the gels, with the bigel showing better efficacy, due to its lower maximal effective dose, compared to the thermosensitive gel.

**Figure 6:**
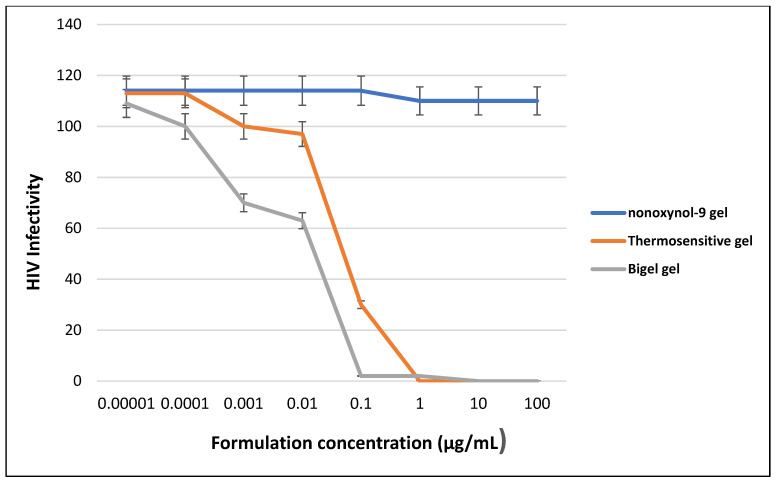
HIV infectivity dose-response curve for the optimal thermosensitive and bigel, containing maraviroc and tenofovir microspheres and nonoxynol-9 gel, incubated with HIV-1 indicator TZM-bl cells at different concentrations (*n* = 5 ± SD). No significant difference between the mean HIV infectivity values of the thermosensitive and bigel (*p* > 0.05).

Furthermore, there was an observed contrasting difference between the response of the negative control (nonoxynol-9 gel), compared to the thermosensitive gel and bigel, as shown in Figure 6.

### 2.6. Enzyme Degradation Assay

Exploiting enzymes as a trigger in the arena of smart drug delivery is valuable because of enzyme specific selectivity for its substrate [16]. Acid phosphatase enzymes are known to hydrolyze phosphate groups [17], and the optimal microspheres in this study were formulated using ionic gelation technique via a crosslinking of chitosan with tripolyphosphate.

The hypothesis that the crosslink formed by tripolyphosphate with chitosan might be hydrolyzed using acid phosphatase enzyme to influence and trigger the release of the drugs encapsulated in the microspheres was examined in this study. The rate and duration of drug release from the microspheres was faster under the influence of acid phosphatase enzyme. This is an indication that there was a breakdown of the polymer matrix to release the drugs faster. The release kinetics for maraviroc transformed from zero-order to first-order model kinetics. This implies that the release kinetics changed from being constant to a concentration-dependent release. The rate of tenofovir release was faster in the presence of the enzyme, resulting in a complete release at the end of 24 h, compared to its gradual release pattern beyond 72 h without enzyme influence. The drug transport mechanism for tenofovir changed from non-Fickian transport system to a super case II transport mechanism. Acid phosphatase enzyme is one of the enzyme compositions of the seminal fluid; therefore, these results suggest the possibility of a breakdown of the microsphere polymer matrix when the seminal fluid encounters the microspheres during sexual intercourse, thus triggering release of the drugs. Researchers have explored the possibility of other enzymes present in the seminal fluid serving as a trigger to facilitate the release of drugs by exploring polymers that can be hydrolyzed in the presence of these enzymes, which has informed the concept of this study. Some researchers [18] demonstrated the ability of hyaluronidase enzyme to hydrolyze the drug–polymer matrix, formulated using hyaluronic acid; a polymer that is hydrolyzed by hyaluronidase.

### 2.7. Evaluation of the Thermosensitive Gel and Bigel

A suitable vehicle to deliver the microspheres into the vagina is desirable and necessary. The goal of developing a microbicide formulation, with prolonged residence time in the vagina, has necessitated the use of gelling agents, such as poloxamers.

The combination of poloxamers 407 and 68, at different ratios, have been exploited for ocular, transdermal, and vaginal drug delivery [19,20].

Micelle formation by poloxamers in aqueous solution is reversible, and this is enhanced by the amphiphilic nature of the copolymers. The mechanism by which temperature sensitive hydrogels achieve thermogelation is either by cooling below the upper critical temperature (UCGT) or heating above the lower critical gelation temperature [21].

The poloxamer combination used in this study transitioned from solution to gel by heating above the lower critical gelation temperature, falling within the range of 33 °C to 37 °C.

The different combinations of pluronics F127 and F68, used for the formulation of the thermosensitive gel in this study, had varying effects on the gelation characteristics of the thermosensitive gel. It was observed that concentrations of PF127 below 2 g did not become viscous, even at high temperatures. The optimal thermosensitive gel showed varying viscosity values at different temperatures. Viscosity increased with increasing temperatures, with a quantum leap in viscosity values between 25 °C and 37 °C (88 to 62,887 cps). At 25 °C, the gel was still in liquid form, which is desired, as this is necessary to allow for easy handling and administration. However, at 37 °C, which is the vaginal temperature, the gel becomes viscous, which is equally desired because the goal is to prevent the gel from leaking from the vagina and combat the challenges of leakage experienced with conventional vaginal gels; this explains the high viscosity at this temperature.

Osmolality is an important parameter for an ideal microbicide gel [22]. Acceptable values below 1000 mOsm/kg are recommended to prevent mucosal damage and epithelial stripping of the vaginal tissues. The thermosensitive gel had an osmolality of 991mOsm/kg, which is within acceptable range. One of the setbacks of the 1% tenofovir gel, used for the CAPRISA 004 study, was the hyperosmolality (3111 mOsm/kg) of the gel [5].

A bigel is ideal for the simultaneous incorporation of hydrophilic and lipophilic drugs, such as tenofovir and maraviroc. The optimal formulated bigel had a pH of 3.65, which favors the protective acidic pH condition of a healthy vaginal environment.

Its viscosity value taken at room temperature was 8840 cps. Bigels are a blend of a highly a viscous organogel and less viscous hydrogel, which results in a gel with good spreadability and retention that should surmount the problems of messiness and leakage associated with previous conventional gels [23].

Osmolality is an important parameter for an ideal microbicide gel. Acceptable values below 1000 mOsm/kg are recommended to prevent mucosal damage and epithelial stripping of the vaginal tissues. The bigel had an osmolality of 628 mOsm/kg, which is within the acceptable range. One of the setbacks of the 1% tenofovir gel, used for the CAPRISA 004 study, was the hyperosmolality (3111 mOsm/kg) of the gel [5,22].

### 2.8. Comparative Biophysical Analysis of the Thermosensitive Gel and Bigel

The two smart gels formulated in this study belong to different category of gels; however, the goal is to design vaginal gels that will combat the challenge of short residence time in the vagina, leakage, and coital dependent administration. Both gels are suitable candidates for vaginal delivery of microbicide but differ, with respect to various rheological parameters and efficacy.

With respect to gel viscosity, which is a function of the residence time and leakage, both gel types demonstrated improved viscosity, compared to regular conventional hydrogels; however, thermosensitive gel has a higher viscosity, with a better tendency for retention in the vagina.

The osmolality values of both gels were below 1000 mOsm/kg, with a lower osmolality value recorded for the bigel, indicating a lower tendency for vaginal mucosa damage and epithelial stripping, compared to the thermosensitive gel.

The pH values of both gels were acidic, as is required for maintaining a conducive vaginal environment, although the pH value of the bigel was lower, compared to the thermosensitive gel. There was an adequate release of tenofovir and maraviroc from the thermosensitive and bigels, with no significant difference in their release profile. Tenofovir release followed Higuchi model kinetics from the thermosensitive and bigel and maraviroc, followed a zero-order kinetics order release from both gels, thus eliciting a similar pattern in the HIV infectivity decline rate.

The thermosensitive and bigel demonstrated suitable properties, which makes them potential appropriate microbicide candidates.

The pre-exposure prophylactic ability of the combined optimal tenofovir and maraviroc microspheres were evaluated via an in-vitro concentration-response procedure, using nonoxynol-9 as the control. Nonoxynol-9 is a spermicidal agent that was previously one of the early nonspecific antiviral agents that were tested as microbicides but failed in clinical trials [24,25].

The HIV infectivity of the thermosensitive gel and bigel, compared with nonoxynol-9, showed a marked difference in the HIV infectivity rate against the CCR5 tropic HIV-1 BaL strain.

The observed continuous decline in HIV infectivity of the microspheres, indicates adequate release of the drugs from the polymer and anti-HIV activity.

In comparing the efficacy of the bigel with the thermosensitive gel, the former appears to have a better potency, as demonstrated in it achieving a lower maximum effective dose than that of the thermosensitive gel. This might be attributed to the fact that bigel accommodates both lipophilic drugs and hydrophilic simultaneously and might allow better release, in comparison to the thermosensitive gel that will favor the release of hydrophilic drugs better than lipophilic drugs. Tenofovir is a hydrophilic drug, while maraviroc is lipophilic; therefore, a system that will favor the release of both drugs should demonstrate better potency as observed.

Ibrahim et al. [26], in a study of drugs incorporated into a hydrogel, organogel, and bigel, were able to demonstrate the potency of the formulation incorporated into the hydrogel, attributing it to the hydrophilicity of the drug, which was favored in the hydrogel.

### 2.9. In-Vitro Cytotoxicity Assay

The safety of any microbicide formulation on vagina cells is an important aspect of microbicide evaluation. A disruption or toxicity on vaginal cells will negatively impact adherence to its use and microbicide acceptance, regardless, of its efficacy. Lackman-Smith et al. [27], evaluated natural product-based microbicides containing lemon, lime, and vinegar, and the study reported the cytotoxicity of this microbicide to HeLa cells. Although this product demonstrated anti-HIV activity, its cytotoxicity did not fulfill the criteria for an ideal microbicide.

Maraviroc and tenofovir microspheres were exposed to HeLa cells, in order to evaluate cell viability and cytotoxicity in-vitro, to assess the effect on these cells over a 48-h period.

Maraviroc and tenofovir microspheres did not show significant signs of cytotoxicity on the HeLa cells at a concentration of 10 μg/mL. The cells showed a percentage viability of 95.3%, upon exposure at this concentration.

The absorbance values, which are a measure of the viable cells, did not have a statistically significant value at this concentration, when compared to the negative control (*p* = 0.054).

Similarly, some studies [28,29] demonstrated the safety of a 1mg/mL tenofovir microsphere suspension on vaginal and epithelial cell lines, which showed no significant reduction in cell viability.

## 3. Conclusions

Vaginal microbicide gels containing maraviroc and tenofovir microspheres were successfully developed, with adequate in-vitro release profiles that suggest good formulation properties.

Maraviroc and tenofovir microspheres have potential for a triggered release by acid phosphatase enzyme present in the seminal fluid, thus serving as a strategic point to prevent HIV transmission, when used by women during heterosexual intercourse. Its efficacy on the HIV-1 BaL virus strain and safety on vaginal lactic acid bacteria and HeLa cells suggests further in vivo studies might pave the way for promising results.

## 4. Materials and Methods

### 4.1. Materials

Poloxamer 407 (Pluronic F127), poloxamer 188 (Pluronic F68), and chitosan were supplied by Dideu Medichem Ltd. (China). Maraviroc was purchased from Shanghai Yudiao Chemistry Technology Co Ltd. (Shanghai, China). Tenofovir was supplied by Macklin Biochemical Co. Ltd. (Shanghai, China). Sodium tripolyphosphate, Span 60, and Hydroxy propyl methyl cellulose were procured from Shanghai Macklin Biochemical Co. Ltd. (Shanghai, China). Soya bean oil was purchased from Sunola Foods Ltd. (Lagos, Nigeria).

Milli-Q water (Milli-Q water purification system, Merck, Germany) was used for all the experiments. All other chemicals were of analytical grade and used as obtained from the suppliers, without further modification.

**Cell lines:** HeLa cells and fetal bovine serum were purchased from Cell Lines Service GmbH, Germany. HIV-1 BaL and TZM-bl cells were obtained from Fisher Bioservices /NIH-ARP German town, MD, USA.

### 4.2. Methods

#### 4.2.1. Microsphere Preparation and Characterization

Maraviroc and tenofovir microspheres were prepared using an ionic gelation technique, whereby chitosan was crosslinked with sodium tripolyphosphate to form microparticle carriers for the drugs. Varying concentrations of chitosan (0.1 and 0.2 g) were dissolved in 10 mL of acetic acid (4% *v*/*v*), with a subsequent addition of 10 mg of the individual antiretroviral drugs. The resultant dispersion was added in droplets into a (2% *w*/*v* and 4 % *w*/*v*) solution of sodium tripolyphosphate, using a 23 G syringe nozzle.

The resultant wet microsphere beads were allowed a curing time of 10 to 15 min, then dried at 45 °C for 12 h. The dried microspheres were further characterized for particle size, encapsulation efficiency, drug excipient compatibility, mucoadhesion, and in-vitro release, with details in [30].

#### 4.2.2. Enzyme Degradation Assay

A phosphatase assay, using the enzyme recombinant human acid phosphatase, was used to analyze the effect of the enzyme on the release of the drugs from the drug–polymer matrix of the microspheres. A combination of seminal fluid simulant (SFS) and vaginal fluid simulant (VFS) were mixed at a ratio of 1:4, and the pH adjusted to 7.1 using 0.1 N HCl. The amount of acid phosphatase enzyme (200 U) present in an average volume of seminal ejaculate was added to 3 mL of the SFS and VFS mixture.

The formulated maraviroc and tenofovir microspheres were each resuspended in 3 mL of a mixture of seminal fluid simulant and vaginal fluid simulant (pH 7.1), and the dispersion was transferred into a 10 mL capacity dialysis membrane (Spectra/Por, Float-A-Lyzer G2, MWCO 3.5-5KD, Spectrum Laboratories Inc., Rancho Dominguez, CA, USA).

The membrane was maintained in a 20 mL release medium (SFS and VFS, pH 7.1), contained in the dialysis tube, and the whole system was placed in a conical flask, then put in a shaking water bath (Julabo SW-21C, Germany) and maintained at a temperature of 37 °C. At periodic time intervals of 0, 1, 3, 6, 24, 48, and 72 h, a 1 mL sample was withdrawn from the release medium in the dialysis tube for analysis. At each point of withdrawal an equivalent volume of the release medium was replaced to maintain sink condition [31]. The amount of maraviroc and tenofovir released was determined using HPLC (Agilent 1200 series, Germany), under the same conditions as described above for encapsulation efficiency. The above procedure was repeated, without addition of the recombinant human acid phosphatase enzyme, and this served as the control.

Mathematical models, which include the zero-order, first-order, Higuchi, and Korsmeyer–Peppas models, were used to interpret the release kinetics of the drugs, and the model with highest coefficient of correlation (R^2^) best describes the pattern of drug release.

A zero-order model describes a process of constant drug release, which is determined by a plot of cumulative percentage drug released versus time (h). A first-order model depicts a process in which drug release is dependent on the drug concentration; it is represented graphically by a plot of the log of cumulative percentage drug remaining against time (h).

Diffusion controlled release is the prime mechanism of drug release for a Higuchi model, which is obtained from a plot of cumulative percentage drug released against square root of time. Furthermore, the Korsmeyer–Peppas model helps to ascertain the type of drug transport mechanism, which is characterized by the *n* value. The coefficient of X in the straight-line equation of the graphs represents (*n*), which depicts the drug transport mechanism, which is usually a range of values [32].

#### 4.2.3. Preparation of Thermosensitive Gel

A citrate buffer of pH 4.5 was prepared by dissolving 42 mg of citric acid and 59 mg of trisodium citrate dihydrate in 1000 mL of Mili Q water, and pH was adjusted to 4.5 using 0.1 N HCl. A combination of the optimal maraviroc and tenofovir microspheres was added to 10 mL of this buffer, and then varying quantities of the polymers PF127 (Poloxamer 407) and PF68 (Poloxamer 188) were added to the dispersion, in a sample bottle, to identify the optimal combination that will give the desired gelation temperature. Methylparaben sodium (0.1%) and propyl paraben sodium (0.01%) were added as preservatives. This dispersion was kept in a refrigerator (Haier Thermocool, HRF350), overnight at 2–8 °C, to dissolve the polymers [5,33]. The resultant solution was assessed for gelation temperature, gelation time, and rheological properties.

#### 4.2.4. Preparation of Bigel

The bigel was prepared from a hydrogel and organogel mix. The hydrogel was prepared by weighing 15 g of hydroxypropyl methylcellulose into 150 mL of Milli-Q water, then allowed to soak and dissolve overnight. The dispersion was stirred for 30 min with a magnetic stirrer, and the pH was adjusted to a pH of 2.01, using 0.1 N HCl. In preparing the organogel varying concentrations of Span 60 were prepared in soya bean oil ranging from 2%, 5%, 10%, 15%, 18%, 20%, and 25%. Tween 80 was added as surfactant. The combination F3, was used for organogel preparation. To prepare 100 g of the organogel, 10 g of Span 60 was weighed into a beaker containing 96 mL of soya bean oil and 1.8 mL of Tween 80. This was placed in a water bath at 60 °C and stirred until there is a homogenous mix. The dispersion was allowed to set at room temperature, after which it is stored at 2–8 °C overnight. The bigel was then prepared by mixing at a ratio of 1:1, 1:2, 2:1, 1:3, and 3:1 of the hydrogel and organogel to form a homogenous mix, with subsequent determination of the optimal gel mix. The tenofovir and maraviroc microspheres were then incorporated into the formulation for the optimal hydrogel and organogel (1:1), respectively, and then combined to formulate the optimal bigel mix [34].

### 4.3. Characterization of the Thermosensitive Gel and Bigel

#### 4.3.1. Gelation Temperature and Time

A 25 mL beaker containing 10 mL of the pluronics dispersion was placed in a water bath, and the probe of a digital thermometer was inserted in the gel. The gel was heated with continuous stirring and temperature monitoring. The temperature at which the dispersion turned into a gel and did not flow upon inversion of the beaker was the gelation temperature. The time interval between when heat was applied and the dispersion became viscous is known as the gelation time [35].

#### 4.3.2. Rheological Study and pH Determination

The viscosity and osmolality of the optimal gel (T2) was evaluated. A viscometer was used to determine the viscosity of T2 gel at varying temperatures. At a speed of 30 rpm, and with a spindle of size of 63, the viscosity of the gel at 4 °C and 25 °C was determined. While at a speed of 6 rpm, and with a spindle size of 64, the viscosity of the gel at 37 °C was a determined. The osmolality of the gel was also analyzed using an osmometer. The pH of the various sample combinations was determined using an Adwa pH/mV and temperature bench top pH meter (AD1040 series, Hungary). After calibration, the probe of the pH meter was inserted into the gel samples, and pH readings were recorded.

#### 4.3.3. In-Vitro Release of Microspheres from the Thermosensitive Gel

The in-vitro release of the drugs from the gels were evaluated using a dialysis membrane. The individual gels containing optimal microspheres of tenofovir and maraviroc were introduced in the dialysis membrane, as well as the procedure described above (in the methodology section) for enzyme degradation assay.

#### 4.3.4. Rheological Study and pH Determination

The viscosity and osmolality of the optimal bigel mix was evaluated. A viscometer (Brookfield viscometer, DV1) was used to determine the viscosity of the gel at a speed of 12 rpm and with a spindle of size of 64, the viscosity of the gel at room temperature was determined. The osmolality of the gel was also analyzed using an osmometer.

The pH of the various bigel sample combinations was determined using an Adwa pH/mV and temperature bench top pH meter (AD1040 series, Hungary). After calibration, the probe of the pH meter was inserted into the gel samples, and pH readings were recorded.

#### 4.3.5. In-Vitro Release of Microspheres from the Bigel

The in-vitro release of the drugs from the bigel were evaluated using a dialysis membrane. The individual bigel containing optimal microspheres of tenofovir and maraviroc were introduced in the dialysis membrane and procedure described in above (in the methodology section) for enzyme degradation assay.

#### 4.3.6. In-Vitro Cytotoxicity of the Microspheres on Vaginal HeLa Cells

The in-vitro cytotoxicity of the microspheres was evaluated using HeLa cells [18]. The cells, contained in a T25 flask, were allowed to grow until about 80% confluence was achieved. The media from the plates was drained using a 10 mL serological pipette and pipette gun (Dragon Med hero plus). A 100 µL solution of Trypsin EDTA (0.25%), at a pH of 7.21, was added to the flask to dislodge the cells. The flask containing the cells was placed in an incubator at 37 °C for 5 min. A 20 mL solution of 5% Dulbecco’s modified Eagle media (DMEM) was added to the cells in the flask, then the whole suspension of cells was collected using a serological pipette into a falcon tube and centrifuged (MRC centrifuge) at 400 *g* for 10 min.

The sedimented cell suspension was collected after decanting the supernatant media. A total of 50 µL of trypan blue was added to 50 µL of the cell suspension, and this was transferred to a hemocytometer. The hemocytometer was viewed under the microscope, and the number of cells in each quadrant of the hemocytometer was counted using a tally counter, and an average of the cell count was determined. The viable cells were determined by the following formula:

(1) Viable cells/mL=Cell count×scaling factor×dilution factor

(Where scaling factor =104 and dilution factor = 2)

Using the formula C_1_V_1_ = C_2_V_2_, the volume of cell suspension (V_1_) needed to achieve a cell suspension V_2_ of 30 mL (capacity of 96-well plates for the test and controls) and 10^5^ cells/mL concentration (C_2_) was calculated from the viable cell/mL of 2.555 × 10^6^ cells/mL (C_1_). V_1_ was calculated to be 1.17 mL. A volume of the cell suspension of 1.17 mL was measured into a plastic trough and made up to 30 mL using 5% DMEM.

A multichannel pipette was used to transfer 100 µL of the cell suspension (at a density of 10^5^ cell/mL) into each well of the 96-well plates, and it was covered, sealed on all sides, and incubated at 37 °C for 24 h.

After incubation, the media was drained from each well, serial dilutions of concentrations of maraviroc and tenofovir microsphere at 1000 μg/mL to 0.1 μg/mL were added in triplicates to the wells containing the cells.

Camptothecin, at a concentration of 1000 μg/mL, was used as the positive control, while the media was used as the negative control. The 96-well plates were covered and sealed, then incubated at 37 °C for 24 h.

After 24 h, the media was drained from each well and 10 µL of water-soluble tetrazolium salt (WST) was added to each well. A Biochrom EZ microplate reader was used to determine the absorbance of the cells, at a wavelength of 450 nm.

The percentage viability was determined from the Equation:

(2) $$\mathrm{Viability}\;(\%)=\frac{\mathrm{Absorbance}\left(\mathrm{test}\right)}{\mathrm{Absorbance}\;\left(\mathrm{control}\right)}\;\times \;100$$

#### 4.3.7. Efficacy Testing

TZM-bl cells assay was described by [20]. TZM-bl cells were plated, and 100 μL of the 10-fold serial dilution of the thermosensitive and bigel-containing maraviroc and tenofovir microspheres, in a 50:50 ratio, was applied. Nonoxynol 9 gel was used as the control. For efficacy testing, 100 μL of medium containing HIV-1BaL, without and with 12% simulated seminal fluid, was added to each well. After 48 h, 100 μL of medium was removed and replaced with 100 μL of Bright-Glo, and the luminescence measured. Inhibition was determined based on deviations from the HIV-1-only or HIV-1/semen controls.

## Figures and Tables

**Table 1:** Cumulative percentage of drug released from microspheres, with and without acid phosphatase enzyme.

Time (h)	Maraviroc (Without Enzymes)	Maraviroc (With Enzymes)	Tenofovir (Without Enzymes)	Tenofovir (With Enzymes)
0	0	0	0	0
1	0	0	30.84	34.88
3	0	0	33.80	40.13
6	0	0	35.60	54.36
9	0	0	39.80	58.86
12	31.02	46.3	42.76	59.66
24	42.31	66.2	49.20	99.65
48	60.65	75.4	79.92	-
72	94.70	100	81.40	-

**Table 2:** Mathematical models for maraviroc and tenofovir release kinetic data.

Mathematical Models	R^2^ Values without Enzyme *	R^2^ Values with Enzyme *
**Maraviroc**		
Zero-order	0.9409	0.8460
First-order	0.8966	0.8922
Higuchi model	0.8953	0.8687
Korsmeyer–Peppas model	0.7543 *n* = 1.1329 **	0.7292 *n* = 1.239 **
**Tenofovir**		
Zero-order	0.8086	0.8475
First-order	0.8590	0.8470
Higuchi model	0.9034	0.9473
Korsmeyer–Peppas model	0.4907 *n* = 0.5973 **	0.5039 *n* = 0.8971 **

* R^2^ = coefficient of correlation and the highest value best describes a drug kinetic model of release. ** Depicts the drug transport mechanism(*n*).

**Table 3:** Composition of a 10 mL thermosensitive gel and characterization results.

Code	PF127 (g)	PF68 (g)	Gelation Temperature* (°C)	Gelation Time * (secs)	pH
T1	2.0	0.08	37.6 ± 0.6	37 ± 1.4	5.69
T2	2.0	0.1	36.4 ± 0.8	36 ± 1.1	5.83
T3	1.8	0.1	No gelation	-	5.97
T4	1.5	0.1	No gelation	-	5.94
T5	2.0	0.2	39.4 ± 1.3	39 ± 1.6	5.84
T6	1.8	0.2	42.6 ± 2.2	54 ± 1.8	5.89
T7	1.5	0.2	No gelation	-	5.97
T8	2.0	-	33.4 ± 0.9	96 ± 1.5	5.02
T9	1.8	-	39.8 ± 0.8	120 ± 2.2	5.89

* Each value is presented as mean ± SD, *n* = 3.

**Table 4:** Characterization for thermosensitive gel containing maraviroc and tenofovir microspheres.

Parameter	Values
pH	5.83
Osmolality	991 mOsm/kg.
Viscosity at 4 °C	24cps
Viscosity at 25 °C	88 cps
Viscosity at 37 °C	62,887 cps
Release kinetic model of maraviroc from the gel	Zero-order (R^2^ = 0.9051)
Release kinetic model of tenofovir from the gel	Higuchi model (R^2^ = 0.9163)

**Table 5:** Formulation chart for varying organogel combinations and gelation characteristics.

Code	Span 60%*w*/*v*	Tween 80 *v*/*v*	Soya Bean Oil %*v*/*v*	Gel Characteristics
F1	2	1	97	No gelation
F2	5	1	94	No gelation
F3	10	1	89	Gelation
F4	15	1	84	Gelation
F5	18	1	81	Gelation
F6	20	1	79	Gelation
F7	25	1	74	Gelation
F8	2	2	96	No gelation
F9	5	2	93	No gelation
F10	10	2	88	Gelation
F11	15	2	83	Gelation
F12	18	2	80	Gelation
F13	20	2	78	Gelation
F14	25	2	73	Gelation

**Table 6:** Varying combinations of organogel–hydrogel mix for bigel formation.

Code	Ratio (Organogel: Hydrogel)	pH of Bigel Mix
T1	1:1	3.65
T2	2:1	4.43
T3	3:1	3.8
T4	4:1	4.8
	**Ratio (Hydrogel : Organogel)**	
T5	1:2	3.5
T6	1:3	3.7
T7	1:4	5.6

**Table 7:** Characterization parameters for the optimal bigel containing Maraviroc and Tenofovir microspheres.

Parameter	Value
pH	3.65
Osmolality	628 mOsm/kg
Viscosity	8840 cps
Release kinetic model of maraviroc from the bigel	Zero-order (R^2^= 0.9431)
Release kinetic model of tenofovir from the bigel	Higuchi model(R^2^ = 0.9206)

**Table 8:** Independent sample *t*-test, comparing the means of absorbance of the test agent and controls to test for significance in cytotoxicity assay.

S/N	Test Agent	N	Viability ** (%)	Mean ± SD (Absorbance)	*p* Value *
1	MVC/TFV (1000 μg/mL)	3	71.2	0.371 ± 0.014	0.001
	Negative control	3	100	0.521 ± 0.01	
2	MVC/TFV (100 μg/mL)	3	83.5	0.435 ± 0.02	0.006
	Negative control	3	100	0.521 ± 0.01	
3	MVC/TFV (10 μg/mL)	3	95.3	0.497 ± 0.011	0.054
	Negative control	3	100	0.521 ± 0.01	
4	MVC/TFV (1 μg/mL)	3	98.1	0.511 ± 0.001	0.069
	Negative control	3	100	0.521 ± 0.01	
5	MVC/TFV (0.1 μg/mL)	3	98.4	0.513 ± 0.002	0.098
	Negative control	3	100	0.521 ± 0.01	
6	MVC/TFV (1000 μg/mL)	3	71.2	0.371 ± 0.014	<0.001
	Positive control	3	13.5	0.071 ± 0.01	
7	MVC /TFV (100 μg/mL)	3	83.5	0.435 ± 0.02	<0.001
	Positive control	3	13.5	0.071 ± 0.01	
8	MVC/TFV (10 μg/mL)	3	95.3	0.497 ± 0.011	<0.001
	Positive control	3	13.5	0.071 ± 0.01	
9	MVC/TFV (1 μg/mL)	3	98.1	0.511 ± 0.001	<0.001
	Positive control	3	13.5	0.071 ± 0.01	
10	MVC/TFV (0.1 μg/mL)	3	98.4	0.513 ± 0.002	<0.001
	Positive control	3	13.5	0.071 ± 0.01	
11	Negative control	3	100	0.521 ± 0.01	<0.001
	Positive control	3	13.5	0.071 ± 0.01	

* Significance level = 0.05. ** 95% confidence interval. Maraviroc = MVC, Tenofovir = TFV.
